# Cardiac *Hamp* mRNA Is Predominantly Expressed in the Right Atrium and Does Not Respond to Iron

**DOI:** 10.3390/ijms24065163

**Published:** 2023-03-08

**Authors:** Maria Bigorra Mir, Edouard Charlebois, Sofiya Tsyplenkova, Carine Fillebeen, Kostas Pantopoulos

**Affiliations:** 1 Lady Davis Institute for Medical Research, Jewish General Hospital, Montreal, QC H3T 1E2, Canada; 2 Department of Medicine, McGill University, Montreal, QC H4A 3J1, Canada

**Keywords:** hepcidin, hemojuvelin, ferroportin, iron metabolism, atria, cardiomyocytes

## Abstract

Hepcidin is a liver-derived hormone that controls systemic iron traffic. It is also expressed in the heart, where it acts locally. We utilized cell and mouse models to study the regulation, expression, and function of cardiac hepcidin. Hepcidin-encoding *Hamp* mRNA was induced upon differentiation of C2C12 cells to a cardiomyocyte-like phenotype and was not further stimulated by BMP6, BMP2, or IL-6, the major inducers of hepatic hepcidin. The mRNAs encoding hepcidin and its upstream regulator hemojuvelin (Hjv) are primarily expressed in the atria of the heart, with ~20-fold higher *Hamp* mRNA levels in the right vs. left atrium and negligible expression in the ventricles and apex. Hjv^−/−^ mice, a model of hemochromatosis due to suppression of liver hepcidin, exhibit only modest cardiac *Hamp* deficiency and minor cardiac dysfunction. Dietary iron manipulations did not significantly affect cardiac *Hamp* mRNA in the atria of wild-type or Hjv^−/−^ mice. Two weeks following myocardial infarction, *Hamp* was robustly induced in the liver and heart apex but not atria, possibly in response to inflammation. We conclude that cardiac *Hamp* is predominantly expressed in the right atrium and is partially regulated by Hjv; however, it does not respond to iron and other inducers of hepatic hepcidin.

## 1. Introduction

Iron is an essential constituent of many cellular proteins, and its deficiency causes adverse effects such as anemia [1] or heart failure [2]. On the other hand, excess iron is detrimental due to its redox reactivity that promotes oxidative stress and leads to tissue injury [3]. Thus, iron overload may cause cardiomyopathy [4] or liver disease (fibrosis, cirrhosis, hepatocellular carcinoma) [5,6]. Iron homeostasis is critical for health and is maintained by elegant mechanisms that operate at the systemic and cellular levels.

Hepcidin is a liver-derived peptide hormone that acts as a master regulator of systemic iron traffic. It operates by binding to the iron exporter ferroportin in target cells, which promotes ferroportin degradation or directly blocks iron efflux into plasma [7]. By inactivating ferroportin in duodenal enterocytes and tissue macrophages, hepcidin inhibits dietary iron absorption and the release of recycled iron from senescent red blood cells, respectively.

Hepcidin expression is regulated by multiple pathways, with BMP/SMAD signaling to be the most prominent [8]. An increase in iron stores triggers the induction of bone morphogenetic protein 6 (BMP6) in liver sinusoidal endothelial cells, which also express BMP2. Secreted BMP6 and BMP2 bind to BMP receptors and the BMP co-receptor hemojuvelin (Hjv) in neighboring hepatocytes to activate the SMAD (small mothers against decapentaplegic) signaling cascade, culminating in transcriptional activation of the hepcidin-encoding *HAMP* gene. Iron-dependent hepcidin induction serves to prevent further iron absorption, while genetic defects in the pathway account for hereditary hemochromatosis, a disease of iron overload [9].

Under inflammatory conditions, liver hepcidin is induced mainly via the IL-6/STAT3 pathway. This mechanism causes inflammatory hypoferremia, an innate immune response that aims to restrict iron access to extracellular pathogens [7]. Aberrant hepcidin induction during chronic inflammation limits iron availability for erythropoiesis and contributes to the “anemia of inflammation” [9].

While liver hepatocytes are the predominant source of circulating hepcidin [10], other cell types and tissues produce hepcidin for local control of iron metabolism [7]. Previous studies documented hepcidin expression in the heart [11,12] and, particularly, in cardiomyocytes [13]. Cardiomyocyte-specific ablation of hepcidin in mice, or substitution of wild-type ferroportin with a hepcidin-resistant mutant, resulted in heart failure due to cardiomyocyte iron deficiency [13]. These data revealed a cell-autonomous function of cardiac hepcidin in the regulation of ferroportin levels and iron metabolism in the heart. Nevertheless, there is evidence for the additional contribution of systemic hepcidin [13,14] but also of other hepcidin-independent iron regulatory mechanisms [14,15].

Herein, we utilized C2C12 cells, wild-type mice, and Hjv^−/−^ mice, a model of hereditary hemochromatosis, to study the regulation, expression, and pathophysiological function of cardiac hepcidin. We show that cardiac hepcidin is primarily expressed in the right atrium of the heart by a mechanism that partially depends on Hjv but not on iron or ligands that activate liver hepcidin.

## 2. Results

### 2.1. Differentiation of C2C12 Cells into a Cardiomyocyte-like Phenotype

**Figure 1:**
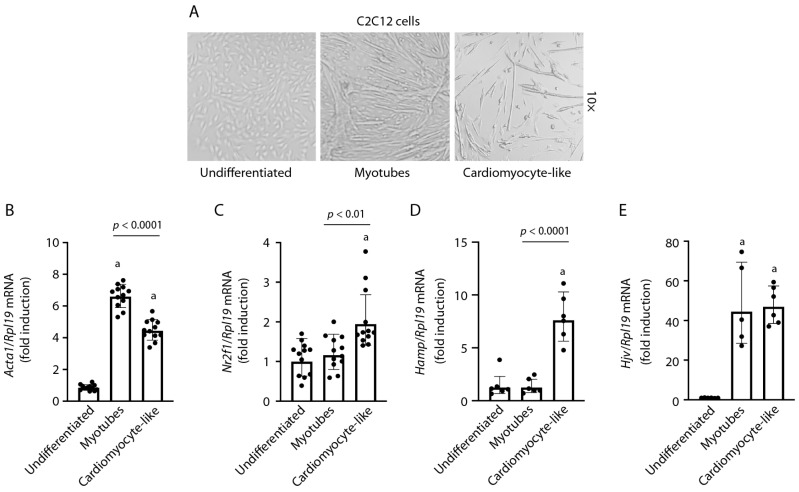
Differentiation of C2C12 myoblasts into a cardiomyocyte-like phenotype. Undifferentiated C2C12 cells were maintained in media containing 20% fetal bovine serum. Differentiation into myotubes was induced by decreasing fetal bovine serum to 2%; addition of 500 nM retinoic acid promoted differentiation into a cardiomyocyte-like phenotype. (**A**) Morphology of undifferentiated C2C12 myoblasts and differentiated myotubes and cardiomyocyte-like cells; magnification: 10×. (**B**–**E**) qPCR analysis of *Acta1*, *Nr2f1*, *Hamp,* and Hjv mRNAs. Gene expression data are represented as geometric mean ± geometric standard deviation. Statistically significant differences (*p* < 0.05) versus undifferentiated cells are indicated by ^a^.

The C2C12 myoblast cell line originates from murine thigh muscle satellite cells obtained after a crush injury [16]. C2C12 myoblasts proliferate in the presence of high serum and differentiate into myotubes under low serum conditions. Thus, they are widely used as a model of skeletal muscle differentiation. Previous work showed that these cells could also acquire cardiomyocyte properties when grown in media preconditioned by primary cardiomyocytes [17]. Considering that retinoic acid is essential for heart development and is utilized for the differentiation of pluripotent stem cells to cardiovascular lineages [18], we hypothesized that a combination of serum deprivation and retinoic acid treatment might promote the differentiation of C2C12 myoblasts toward cardiomyocytes. In fact, C2C12 cells differentiated in the presence of retinoic acid exhibited a distinct morphology devoid of mature myotubes, which is consistent with a cardiomyocyte-like phenotype (Figure 1A). As expected, the differentiation of C2C12 myoblasts into myotubes correlated with the induction of *Acta1* mRNA that encodes α-actin, an essential component of skeletal muscle sarcomeres (Figure 1B). *Acta1* mRNA expression was significantly reduced in C2C12 cells differentiated in the presence of retinoic acid as compared to myotubes, in line with cardiomyocyte differentiation. This was associated with the induction of *Nr2f1* mRNA (Figure 1C), a cardiomyocyte marker [19]. Thus, we refer to these cells as “C2C12 cardiomyocyte-like cells”.

The levels of hepcidin-encoding *Hamp* mRNA were very low in undifferentiated C2C12 myoblasts, and this persisted following C2C12 cell differentiation into skeletal muscle myotubes. However, expression of *Hamp* mRNA was induced at least 5-fold upon C2C12 cell differentiation toward cardiomyocytes (Figure 1D). On the other hand, the upstream *Hamp* regulator Hjv was induced in both myotube and cardiomyocyte lineages (Figure 1E).

### 2.2. Hamp mRNA Is Induced during C2C12 Cardiomyocyte Differentiation and Does Not Respond to BMP6, BMP2, or IL-6

**Figure 2:**
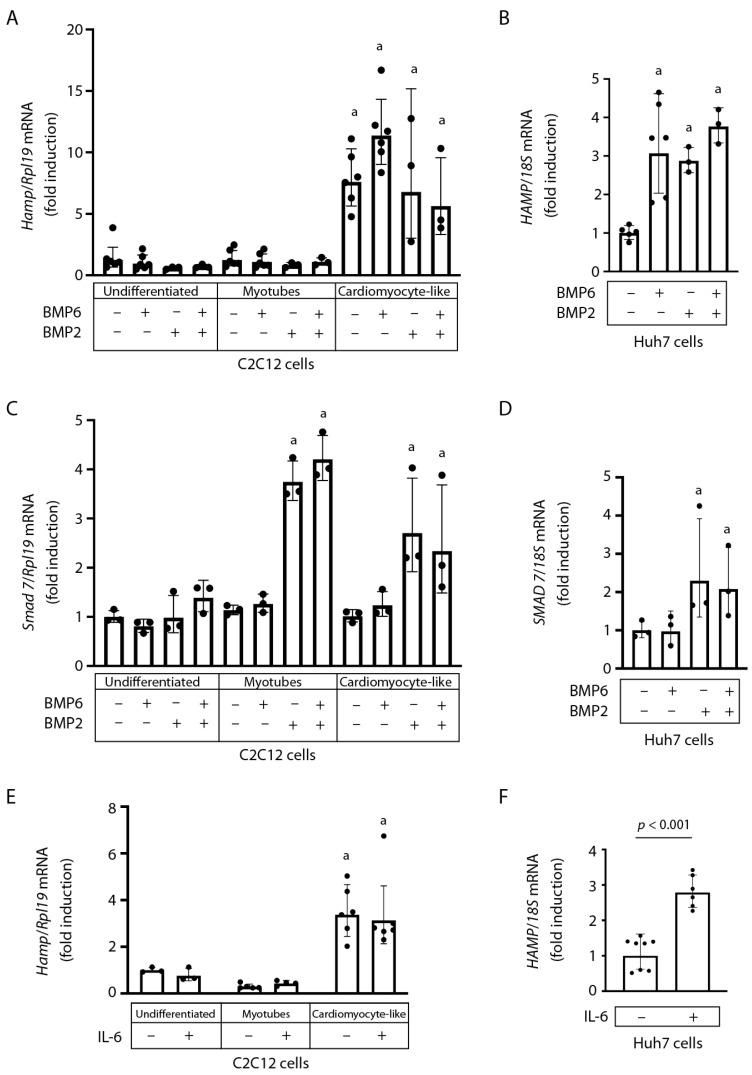
Expression of *Hamp* mRNA in C2C12 cardiomyocyte-like cells does not respond to major inducers of hepatic hepcidin. C2C12 cells, grown as described in Figure 1, and Huh7 hepatoma cells were treated for 4 h with 5 ng/mL BMP6 and/or BMP2, 20 ng/mL murine IL-6 (C2C12 cells), or 10 ng/mL human IL-6 (Huh7 cells). Gene expression was analyzed by qPCR. (**A**–**D**) *Hamp* (**A**), *HAMP* (**B**), *Smad7* (**C**), and *SMAD7* (**D**) mRNA levels following BMP6 and/or BMP2 treatments. (**E**,**F**) *Hamp* (**E**) and *HAMP* (**F**) mRNA levels following treatments with murine or human IL-6, respectively. Gene expression data are represented as geometric mean ± geometric standard deviation. Statistically significant differences (*p* < 0.05) versus undifferentiated C2C12 or untreated Huh7 cells are indicated by ^a^.

Interestingly, *Hamp* mRNA did not respond to treatment with the potent inducers of hepatic hepcidin BMP6 or BMP2 in either undifferentiated or differentiated C2C12 cells (Figure 2A). As expected, BMP6 and BMP2 treatment, alone or in combination, induced *HAMP* mRNA in human Huh7 hepatoma cells (Figure 2B).

The BMP6 and BMP2 treatments did not affect the expression of *Smad7* mRNA in undifferentiated C2C12 cells (Figure 2C), while BMP2 appropriately stimulated the expression of this downstream marker of SMAD signaling in Huh7 cells (Figure 2D). Interestingly, only BMP2 but not BMP6 induced *Smad7* mRNA in differentiated C2C12 cells (Figure 2C).

The inflammatory cytokine IL-6 could not significantly activate *Hamp* mRNA in C2C12 cardiomyocyte-like cells (Figure 2E), even though it appropriately induced *HAMP* mRNA in Huh7 cells (Figure 2F). The above cell culture data suggest that hepatic and cardiac hepcidin are regulated by distinct mechanisms.

### 2.3. Cardiac Hamp mRNA Is Predominantly Expressed in the Right Atrium and Only Partially Depends on Hjv

**Figure 3:**
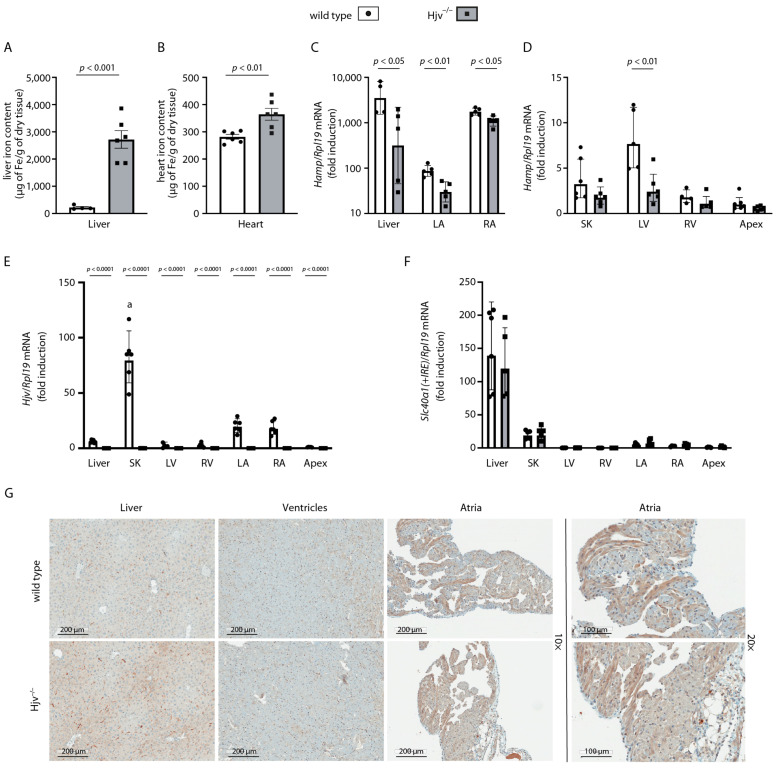
Expression of cardiac *Hamp* mRNA is largely compartmentalized in the right atrium and is only modestly impaired by Hjv deficiency. Nine-week-old male wild-type and isogenic Hjv^−/−^ mice (*n* = 5–6 per genotype) were sacrificed. Livers and hearts were obtained and processed for quantification of iron content, qPCR analysis, and immunohistochemistry. For qPCR analysis, the hearts were previously dissected into left atria (LA), right atria (RA), left ventricles (LV), right ventricles (RV), and apexes. Skeletal muscle (SK) tissue samples were also obtained and used for qPCR analysis. (**A**) Liver iron content; (**B**) Heart iron content; (**C**) *Hamp* mRNA in the liver, LA, and RA; (**D**) *Hamp* mRNA in SK, LV, RV, and apex; (**E**) Hjv mRNA in the liver, SK and heart chambers; (**F**) ferroportin-encoding *Slc40α1*(+*IRE*) mRNA in the liver, SK and heart chambers; (**G**) Immunohistochemical detection of ferroportin in the liver, ventricles, and atria of wild-type and Hjv^−/−^ mice; magnifications: 10× and 20×. Tissue iron data (**A**,**B**) are represented as mean ± SEM, while gene expression data (**C**–**F**) are represented as geometric mean ± geometric standard deviation. In (**E**), statistically significant differences (*p* < 0.05) versus liver Hjv expression are indicated by ^a^.

Inactivation of the *HJV* gene causes juvenile hemochromatosis, an early onset variant of hereditary hemochromatosis [20], and Hjv^−/−^ mice recapitulate the iron overload phenotype [21,22]. We utilized Hjv^−/−^ mice and isogenic wild-type controls to explore the impact of Hjv ablation on cardiac hepcidin expression and iron metabolism. As expected, Hjv^−/−^ mice manifested iron overload in the liver (Figure 3A) and the heart (Figure 3B), which was associated with >10-fold suppression of liver *Hamp* mRNA (Figure 3C).

When we tried to quantify cardiac hepcidin using randomly selected heart tissue sections, we noticed an unusual experimental variability. This prompted us to systematically analyze *Hamp* mRNA levels in dissected chambers of the heart. The data in Figure 3C demonstrate that cardiac *Hamp* mRNA is preferentially and highly expressed in the right atrium. In wild-type mice, *Hamp* mRNA levels in the right atrium were almost comparable, yet ~50% lower, to those in the liver. Meaningful *Hamp* mRNA expression was also evident in the left atrium; nevertheless, it was ~20-fold lower compared to the right atrium. Hjv ablation profoundly (~11-fold) suppressed *Hamp* mRNA in the liver, as expected, but only modestly (~35%) reduced it in both atria of the heart. Notably, *Hamp* mRNA expression in the right and left ventricles and the apex of the heart was minute, as in skeletal muscles (Figure 3D). On the other hand, Hjv mRNA was much more abundant in skeletal muscles vs. the liver of wild-type mice, in line with earlier findings [20], and was also preferentially expressed in the atria but not the ventricles or apex (Figure 3E). These data reveal that cardiac *Hamp* mRNA is highly compartmentalized in the right atrium, and its expression is less dependent on Hjv compared to liver hepcidin.

*Slc40α1* mRNA, encoding the hepcidin target ferroportin, is expressed at much higher levels in the liver vs. the chambers of the heart and appears to be slightly enriched in the atria vs. the ventricles in both wild-type and Hjv^−/−^ mice (Figure 3F). Immunohistochemical analysis showed robust ferroportin staining in the liver and atrial sections, with a weaker signal in the ventricles (Figure 3G). Ferroportin expression appeared higher in the liver and atria of Hjv^−/−^ vs. wild-type mice; the differences in the atria are more visible in the 20× magnification (Figure 3G, right). Stronger expression of cardiac ferroportin in Hjv^−/−^ vs. wild-type mice has also been documented by Western blot analysis of whole heart tissues [14]. Taken together, our findings are consistent with an increased iron efflux potential in atrial vs. ventricular cardiomyocytes, which is further enhanced under conditions of Hjv deficiency.

### 2.4. Atrial Hamp mRNA Does Not Respond to Dietary Iron Manipulations in Wild-Type Mice

**Figure 4:**
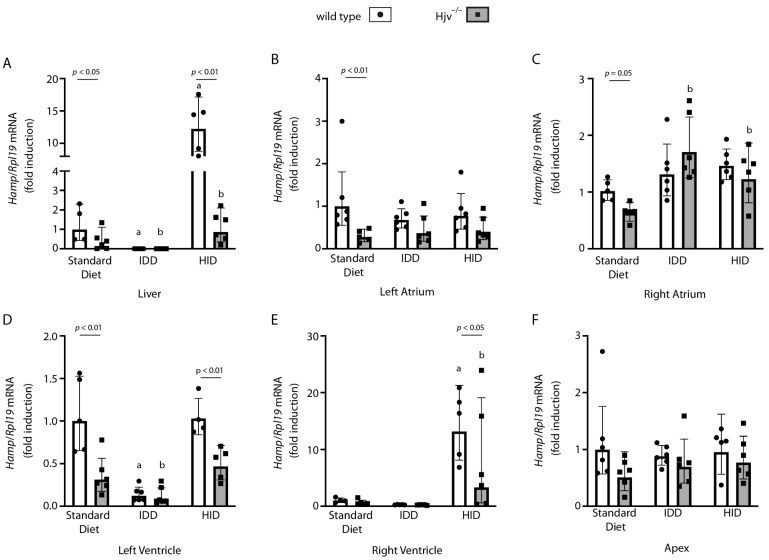
Dietary iron manipulations do not affect atrial *Hamp* mRNA expression in wild-type mice. Ten-week-old male wild-type and isogenic Hjv^−/−^ mice (*n* = 5–6 per group) were fed a standard diet, an iron-deficient diet (IDD), or a high-iron diet (HID) for 6 weeks. At the endpoint, livers were harvested, and hearts were dissected into chambers. The tissue samples were used for qPCR analysis. *Hamp* mRNA was quantified in: (**A**) liver; (**B**) left atrium; (**C**) right atrium; (**D**) left ventricle; (**E**) right ventricle; and (**F**) apex. Data are represented as geometric mean ± geometric standard deviation. Statistically significant differences (*p* < 0.05) versus values from wild-type and Hjv^−/−^ mice on standard diet are indicated by ^a^ and ^b^, respectively.

Expression of liver hepcidin is regulated by body iron stores via the BMP/SMAD pathway [8]. To explore whether cardiac hepcidin is likewise iron-regulated, wild-type and Hjv^−/−^ mice were fed a high-iron diet (HID) or an iron-deficient diet (IDD) for 6 weeks. These dietary manipulations resulted in expected changes in the iron content of the liver, and atria and ventricles of the heart (Figure S1). They also led to appropriate induction or suppression of liver *Hamp* mRNA, respectively, in both genotypes (Figure 4A), yet at a lower scale in Hjv^−/−^ mice, as previously reported [22]. However, *Hamp* mRNA levels in the left (Figure 4B) and right (Figure 4C) atrium of wild-type mice were largely unaffected. Surprisingly, *Hamp* mRNA was slightly induced in the right (Figure 4C) but not left (Figure 4B) atrium of Hjv^−/−^ mice following either HID or IDD intake; the biological significance of this is unclear. Ventricular hepcidin appeared more responsive to iron. Thus, IDD intake suppressed *Hamp* mRNA in the left ventricle (Figure 4D), while HID induced *Hamp* mRNA in the right ventricle (Figure 4E) of both wild-type and Hjv^−/−^ mice. Hepcidin expression in the apex was not affected by dietary iron (Figure 4F). We also attempted to detect the expression of hepcidin peptide by immunohistochemistry; however, the results were inconclusive (Figure S2). Thus, our data suggest that cardiac *Hamp* mRNA, which is predominantly expressed in the right atrium and, to some extent, in the left atrium, is not regulated by iron.

### 2.5. Myocardial Infarction Studies in Wild-Type and Hjv^−/−^ Mice

**Figure 5:**
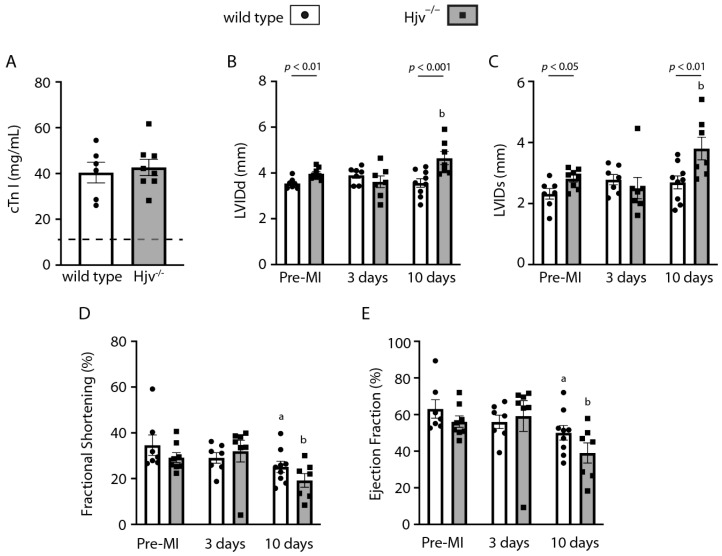
Hjv deficiency has minimal functional implications in the heart. Eleven-week-old male wild-type and isogenic Hjv^−/−^ mice (*n* = 7–9 per group) were subjected to myocardial infarction (MI) surgery. Echocardiography was performed in all animals before as well as 3 and 10 days after the procedure. (**A**) Plasma cardiac troponin (cTn I) levels 24 h after surgery; basal cTn I levels in both genotypes before surgery are indicated by a dotted line. (**B**) Left ventricular internal diameter end diastole (LVIDd); (**C**) left ventricular internal diameter end systole (LVIDs); (**D**) fractional shortening; (**E**) ejection fraction. Data are represented as mean ± SEM. Statistically significant differences (*p* < 0.05) versus values from wild-type and Hjv^−/−^ mice before surgery (pre-MI) are indicated by ^a^ and ^b^, respectively.

We explored whether iron overload due to Hjv and systemic hepcidin deficiency affects heart function following injury. Τo this end, wild-type and Hjv^−/−^ mice were subjected to myocardial infarction surgery. Within 24 h after the procedure, circulating cardiac troponin (cTn I) levels were increased ~3-fold in both genotypes (Figure 5A), indicating a comparable infarct size. Myocardial infarction is associated with alterations in the left ventricular structure, which contribute to the development of heart failure [23,24]. Echocardiography validated loss of contractility and function of the left ventricle in wild-type mice 10 days after surgery (Figure S3). While no significant functional alterations were noted within 3 days, at the 10th day post-surgery LVIDd and LVIDs were significantly increased in Hjv^−/−^ mice, and concomitantly fractional shortening and ejection fraction were significantly decreased in both genotypes (Figure 5B–E). This indicates the impairment of heart function due to the dilation of the left ventricle.

Comparison among genotypes showed significant differences before and 10 days after surgery in LVIDd (*p* < 0.01 and *p* < 0.001, respectively) and LVIDs (*p* < 0.05 and *p* < 0.01, respectively) of wild-type and Hjv^−/−^ mice. These were associated with a trend for decreased fractional shortening and ejection fraction; nevertheless, significance was not reached. Our data suggest that Hjv deficiency only modestly impairs heart function and recovery following myocardial infarction.

**Figure 6:**
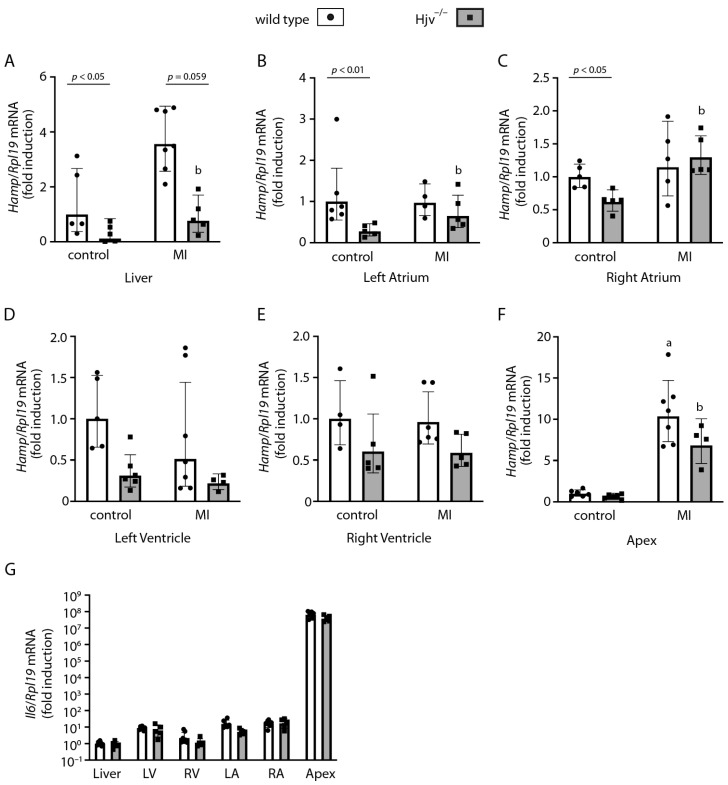
Myocardial infarction promotes delayed *Hamp* induction in the liver and the apex of the heart, possibly in response to an inflammatory response in the apex. Mice described in Figure 5 were sacrificed 2 weeks following myocardial infarction surgery. Livers were harvested, and hearts were dissected into chambers. The tissue samples were used for qPCR analysis. *Hamp* mRNA was quantified in: (**A**) liver; (**B**) left atrium; (**C**) right atrium; (**D**) left ventricle; (**E**) right ventricle; and (**F**) apex. *Il6* mRNA was also quantified in these tissues (**G**). Data are represented as geometric mean ± geometric standard deviation. Statistically significant differences (*p* < 0.05) versus values from control wild-type and Hjv^−/−^ mice (not subjected to surgery) are indicated by ^a^ and ^b^, respectively.

Finally, we monitored hepcidin expression in the liver and heart chambers at the endpoint of the experiment. Liver *Hamp* mRNA was significantly induced in wild-type and to a smaller extent also in Hjv^−/−^ mice 2 weeks after surgery (Figure 6A). Expression of cardiac *Hamp* mRNA in the atria and the ventricles were not affected by the procedure in wild-type mice but appeared slightly increased in the atria of Hjv^−/−^ mice (Figure 6B–E). Nevertheless, *Hamp* mRNA was remarkably induced ~10-fold in the apex of both wild-type and Hjv^−/−^ mice (Figure 6F). This correlated with concomitant dramatic induction of *Il6* mRNA in the apex (Figure 6G), indicating inflammation. In any case, *Hamp* mRNA in the right atrium, which represents the bulk of cardiac hepcidin, was expressed at baseline 2 weeks after myocardial infarction surgery, at least in wild-type mice.

## 3. Discussion

The heart is a major site of extrahepatic hepcidin expression. It is well established that cardiac hepcidin cannot substitute systemic liver-derived hepcidin [10] but rather acts locally [13]. Nevertheless, mechanisms underlying the expression, regulation, and function of cardiac hepcidin remain poorly defined. The scarcity of mechanistic studies may be related to the lack of appropriate cell culture models. Herein, we developed such a model and used it to explore the effects of known inducers of hepatic hepcidin on cardiac.

Thus, C2C12 cells were subjected to differentiation in the presence of retinoic acid. The rationale for this was the ability of retinoic acid to stimulate heart development and differentiation of cardiovascular lineages from pluripotent stem cells [18]. We used a relatively high concentration of retinoic acid (500 nM), which favors atrial specification [19]. Under these conditions, the C2C12 cells acquired a cardiomyocyte-like phenotype with distinct morphological features compared to differentiated C2C12 myotubes (Figure 1A) and expressed the *Nr2f1* marker of atrial cardiomyocytes (Figure 1C). Importantly, they also expressed *Hamp* (Figure 1D) and its upstream regulator Hjv (Figure 1E). *Hamp* mRNA was not induced during myotube differentiation, in line with the lack of significant hepcidin expression in skeletal muscles [11,20]. By contrast, Hjv mRNA was upregulated following C2C12 cell differentiation into either myotubes or cardiomyocytes. Hjv mRNA is highly expressed in skeletal muscles and at lower levels in the heart [20]; it is also known to be induced during the differentiation of C2 [25] or C2C12 [26] myoblasts into myotubes.

The data in Figure 2 show that in C2C12 cardiomyocyte-like cells, *Hamp* mRNA does not respond to treatments with major inducers of hepatic hepcidin such as BMP6, BMP2, or IL-6. These ligands appropriately induced *HAMP* mRNA in human Huh7 hepatoma cells. Activation of the SMAD signaling cascade, as reflected in *Smad7* mRNA induction, was observed in both C2C12 myotubes and cardiomyocyte-like cells in response to BMP2 but not BMP6. This is consistent with the reported capacity of BMP2 to promote C2C12 cell differentiation into an osteoblastic lineage [27]. BMP6 also failed to induce *SMAD7* mRNA in Huh7 cells (Figure 2D). This may be related to the relatively low dose used in the experiment, considering that in primary murine hepatocytes, a clear induction of *Smad7* was obtained with 5 times higher BMP6 concentrations [28]. The data acquired with the C2C12 cell culture model suggest that cardiac and hepatic hepcidin are regulated by different ligands. Nevertheless, the unresponsiveness of cardiac hepcidin to BMP6, BMP2, and IL-6 should be further validated by experiments in primary atrial cardiomyocytes.

We utilized wild-type and Hjv^−/−^ mice to explore the function of cardiac hepcidin but also to assess the role of Hjv on cardiac hepcidin expression and regulation. Hjv^−/−^ mice represent a model of hemochromatosis characterized by iron overload that is severe in the liver and relatively modest in the heart (Figure 3A,B and Figure S1). In preliminary experiments with wild-type mice, we noticed that *Hamp* mRNA levels were notoriously variable in random heart tissue preparations, in agreement with a recent report [14]. A similar analysis in carefully dissected heart chambers revealed that *Hamp* mRNA is highly compartmentalized and predominantly expressed in the right atrium at levels approaching but not reaching those in the liver (Figure 3C). Noteworthy, yet ~20-fold lower expression of *Hamp* mRNA was also evident in the left atrium (Figure 3C), while its levels in the ventricles and the apex were negligible (Figure 3D). It should be emphasized that the first evidence for preferential and high hepcidin expression in the right atrium was provided by Pigeon et al. more than 20 years ago in one of the landmark publications identifying hepcidin as the iron regulatory hormone [11]. Data from dot-blot analysis of *HAMP* mRNA in human tissues and cell lines presented in this paper clearly show a strong signal in the right atrium, which in the text is apparently erroneously referred to as the “left atrium”.

Conceivably, the high expression of hepcidin in atrial cardiomyocytes serves to prevent excessive iron efflux from these cells in an autocrine manner. Our data also show higher expression of the hepcidin target ferroportin in the atria vs. the ventricles (Figure 3F,G). This indicates a propensity for iron loss, which could be protective against the toxicity of iron overload. On the other hand, retention of sufficient iron would be crucial for energy metabolism and is congruous with the high mitochondrial density of atrial cardiomyocytes to support their energy-demanding activities, such as myofilament force generation during contractions [29]. Atrial muscles produce faster contractions than ventricular and account for heart rate and beat-to-beat adaptations [29]. Considering that iron deficiency impairs mitochondrial oxidative phosphorylation [30,31], an inability of atrial cardiomyocytes to retain iron would be detrimental. In fact, mice with cardiomyocyte-specific ablation of *Hamp* develop fatal heart failure due to contractile and metabolic dysfunction, which cannot be rescued by endogenous systemic hepcidin [13]. Similar results were obtained in mice with a targeted disruption of the *Tfrc* gene encoding the iron importer transferrin receptor 1 in the heart [32]. Interestingly, it has been proposed that cardiac hepcidin is induced by hypoxia [12,33]. This would be consistent with the higher *Hamp* mRNA expression in the right vs. left atrium, as cardiomyocytes in this area are in direct contact with deoxygenated blood.

Dietary iron manipulations did not affect *Hamp* expression in the atria of wild-type mice, while, as expected, they had a strong impact on liver *Hamp* (Figure 4A–C). Moreover, Hjv ablation only modestly affected *Hamp* mRNA in the heart, even though it profoundly suppressed it in the liver. These findings demonstrate that *Hamp* expression in the liver and heart is regulated by tissue-specific mechanisms, corroborating the data obtained with the C2C12 cell culture model. It should, however, also be noted that the liver accumulates a considerably higher excess of iron compared to the heart atria or ventricles in response to either genetic or dietary iron overload (Figure S1).

Some iron-dependent regulation of *Hamp* was observed in the ventricles (Figure 4D,E). This may explain the previously reported iron regulation of *Hamp* mRNA in iron-manipulated mice and primary cardiomyocyte cultures [13], assuming that the tissue or cell preparations were enriched in more abundant ventricular cardiomyocytes. Lakhal-Littleton et al. also showed that cardiac hepcidin is subjected to additional post-transcriptional regulation; thus, mature hepcidin accumulates under iron deficiency following cleavage of pro-hepcidin by the pro-protein convertase furin, despite *Hamp* mRNA suppression [13]. Unfortunately, we were unable to reliably assess hepcidin levels in the heart due to technical limitations (Figure S2). Nevertheless, it is tempting to speculate that hypoxic induction of furin [34] in the poorly oxygenated right atrium would further enhance local hepcidin expression.

In rat models of myocardial infarction, *Hamp* mRNA was found induced in ischemic and non-ischemic myocardial biopsies within 6 h [35], and distant from the infarct cardiac apical sections 7 weeks following coronary ligation [36]. In our study with mice, *Hamp* mRNA was analyzed in the heart chambers 2 weeks following surgery. The late endpoint was chosen to monitor the impact of Hjv ablation on the recovery of cardiac function following injury. Under these conditions, *Hamp* mRNA levels were at baseline in the atria and ventricles of wild-type mice and appeared slightly induced in the atria of Hjv^−/−^ mice (Figure 6B–E). Notably, *Hamp* mRNA was ~10-fold induced in the apex in both genotypes, and this response correlated with profound induction of *Il6* mRNA (Figure 6F,G). Presumably, the inflammatory response is related to the surgical procedure, where ligation of the left anterior descending artery stops the blood supply to this part of the heart. Secretion of IL-6 into the bloodstream could explain the observed induction of liver *Hamp* mRNA (Figure 6A). Nevertheless, a limitation of the study design is that the control animals did not undergo a sham operation, which could also promote inflammatory responses.

On a final note, Hjv^−/−^ mice exhibited relatively mild functional defects in the heart before and after myocardial infarction surgery compared to wild-type animals (Figure 5), despite cardiac iron overload. Their phenotype is reminiscent of that of Hamp^−/−^ mice, which likewise accumulate excess iron in the heart due to systemic hepcidin deficiency [37]. On the other hand, mice with tissue-specific ferroportin ablation in cardiomyocytes develop severe cardiac dysfunction with relatively milder iron overload in the heart. However, iron loading in these animals is targeted to cardiomyocytes, while in Hamp^−/−^ mice, excess iron is distributed to other cell types of the heart [37]. Thus, it appears that the iron burden in cardiomyocytes of Hjv^−/−^ mice is not particularly harmful.

In conclusion, we showed that cardiac *Hamp* is highly compartmentalized in the right atrium and does not respond to iron and known inducers of hepatic *Hamp,* such as BMP6, BMP2, or IL-6. However, its expression is partially dependent on Hjv, which raises the possibility of the involvement of other BMP ligands. These remain to be identified in future studies. Likewise, the pathophysiological significance of our findings awaits to be established in further investigations.

## 4. Materials and Methods

### 4.1. Cell Culture

Murine C2C12 myoblasts and human Huh7 hepatoma cells were cultured in Dulbecco’s modified Eagle’s medium supplemented with 20% or 10% fetal bovine serum, respectively, non-essential amino acids, 100 U/mL penicillin and 100 μg/mL streptomycin. The medium was changed every 2–3 days, and the cells were passaged before reaching 80% confluence. For differentiation into myotubes, C2C12 cells were cultured for 2 weeks in media containing 2% fetal bovine serum. For differentiation into a cardiomyocyte-like lineage, C2C12 cells were cultured for 1 week in media containing 2% fetal bovine serum and supplemented with 500 nM retinoic acid. Where indicated, the cells were treated with the following recombinant proteins: 5 ng/mL human/mouse BMP6 and/or BMP2 (R&D Systems, Minneapolis, MN, USA), 20 ng/mL murine IL-6 (Cell Signaling, Danvers, MA, USA), or 10 ng/mL human IL-6 (Sigma-Aldrich, St. Louis, MI, USA).

### 4.2. Animals

Wild-type and isogenic Hjv^−/−^ mice in C57BL/6 background [22] were housed in macrolone cages (up to 5 mice/cage, 12:12 h light-dark cycle: 7 am–7 pm; 22 ± 1 °C, 60 ± 5% humidity). The animals were fed a standard rodent diet containing 200 ppm iron (Teklad Global 18% protein 2018) or, when indicated, iron-deficient (TD.80396; 2–6 ppm iron) or high-iron diets (TD.09521; 2% carbonyl iron). At the endpoints, the mice were sacrificed by CO_2_ inhalation. Liver, skeletal muscle, and heart tissues were dissected and used for biochemical and histological analysis.

### 4.3. Myocardial Infarction Surgery

Nine-week-old mice were anesthetized with 2.5% isoflurane and intubated at the surgery core facility of the Lady Davis Institute. Once stable, the mice were placed in a supine position on a heated table, and the chest cavity was opened. A 7-0 silk suture was used to ligate the left anterior descending coronary artery. Paling of the apical heart (bottom area) verified the infarction. The chest was then closed, and the mice were placed in an incubator until recovery. To reduce pain, the animals were given low-release buprenorphine at the beginning of the surgery, which is effective for 3 days. A serum sample was taken 24 h after surgery from the lateral saphenous vein and initially stored at −20 °C. Cardiac troponin I (cTn I) was subsequently quantified by all serum samples by using an ELISA kit (Novus Biologicals, Littleton, CO, USA), according to the manufacturer’s recommendations. All mice were sacrificed two weeks after surgery.

### 4.4. Echocardiography

Mice were subjected to echocardiography before, as well as 3 and 10 days after myocardial infarction surgery. The procedure was performed under light isoflurane anesthesia (3% isoflurane, 2 L/min O_2_) using a VEVO 3100 ultrasound machine with a center frequency 20 MHz MX250S transducer. The mice were secured lightly in the decubitus dorsal position on a warming pad to maintain normothermia. Five beats were averaged for each measurement. Generally, the heart was first imaged in the two-dimensional (2D) mode in the parasternal long-axis view. The transducer was rotated 45° counterclockwise to obtain a 2-D short-axis view of the mid-left ventricle (LV) at the level of the papillary muscles. Images were used to measure wall thickness and chamber dimensions. Echo parameters, including left ventricular internal diameter end diastole (LVIDd) and left ventricular internal diameter end systole (LVIDs), were determined using VEVO 3100 software. The heart rate (HR) was calculated from two consecutive R-R intervals. Ejection fraction (EF) and fractional shortening (FS) were calculated according to previous literature [38,39] as follows:

$$\mathrm{EF}\;\left(\%\right)=\frac{\left(\mathrm{LVDA}-\mathrm{LVSA}\right)}{\mathrm{LVSA}}\times 100$$

where LVDA is the LV diastolic area and LVSA is the LV systolic area

$$\mathrm{FS}\;\left(\%\right)=\frac{\left(\mathrm{LVIDd}-\mathrm{LVIDs}\right)}{\mathrm{LVIDd}}\times 100$$

Since heart rate correlates with EF and FS, a drop or rise can alter the systolic function assessment [23]. Therefore, only acquisitions with a heart rate between 500 and 550 beats per minute were included in this study.

### 4.5. qPCR Analysis

Total RNA was isolated from harvested cells or mouse tissues using the RNeasy kit and the RNeasy Fibrous Tissue Kit (Qiagen, Toronto, ON, Canada). RNA was quantified with NanoDrop 1000 Spectrophotometer, and its quality was assessed by determining the 260/280 nm absorbance ratios. An RNA aliquot (1 μg) was then reverse transcribed using the OneScript Plus cDNA synthesis kit (Applied Biological Materials Inc., Richmond, BC, Canada). qPCR was performed on a 7500 Fast Real-Time PCR System with the SensiFAST SYBR Lo-ROX Kit from Meridian Bioscience, using gene-specific primers (Table S1). Relative mRNA expression was calculated by the 2^−ΔΔCt^ method [40]. All experiments were normalized using ribosomal protein L19 (*Rpl19*) or 18S ribosomal RNA (*RPS18*) as housekeeping genes for mouse and human samples, respectively.

### 4.6. Iron Assays

Non-heme iron content in the liver and heart was quantified by the ferrozine assay as previously described [41]. Histological detection of iron deposits in the liver was performed by Perls staining, while iron deposits in the atria and ventricles of the heart were visualized by diaminobenzidine-enhanced Perls staining [42].

### 4.7. Immunohistochemistry

Tissue specimens were fixed in 10% buffered formalin and embedded in paraffin. Samples were cut at 4 µm, placed on SuperFrost/Plus slides (Fisher Scientific, Ottawa, ON, Canada), and dried overnight at 37 °C. The slides were then loaded onto the Discovery XT Autostainer (Ventana Medical System, Oro Valley, AZ, USA) for automated immunohistochemistry. Slides underwent deparaffinization and heat-induced epitope retrieval. Immunostaining was performed by using rabbit polyclonal antibodies against ferroportin ([43]; 1:500 diluted) or hepcidin (Abcam, Cambridge, UK; # ab30760; 1:25 diluted) and an appropriate detection kit (Omnimap rabbit polyclonal HRP, #760-4311 and ChromoMapDAB #760-159; Roche Diagnostics, Mississauga, ON, Canada). Negative controls were performed by the omission of the primary antibody. Slides were counterstained with hematoxylin for four minutes, blued with Bluing Reagent for four minutes, removed from the autostainer, washed in warm soapy water, dehydrated through graded alcohols, cleared in xylene, and mounted with Permount (Fisher Scientific, Ottawa, ON, Canada).

### 4.8. Statistical Analysis

Quantitative data were expressed as mean ± standard error of the mean (SEM). Statistical analysis was performed using the unpaired Student’s *t*-test or 2-way ANOVA (with Tukey’s multiple comparisons test) with the Prism GraphPad software (version 9.3.1). A probability value of *p* < 0.05 was considered statistically significant.

## Data Availability

All data are contained within the manuscript and Supplementary Materials.

## Supplementary Materials

The following supporting information can be downloaded at: https://www.mdpi.com/article/10.3390/ijms24065163/s1.
