# Supplementary material for: Cardiac Hamp mRNA Is Predominantly Expressed in the Right Atrium and Does Not Respond to Iron
Source: Int J Mol Sci. 2023 Mar 8;24(6):5163. doi: 10.3390/ijms24065163 (PMC10049151; doi:10.3390/ijms24065163)
Supplement: Supplementary file 1 [file ijms-24-05163-s001.zip › ijms-2208971-figures.pdf]

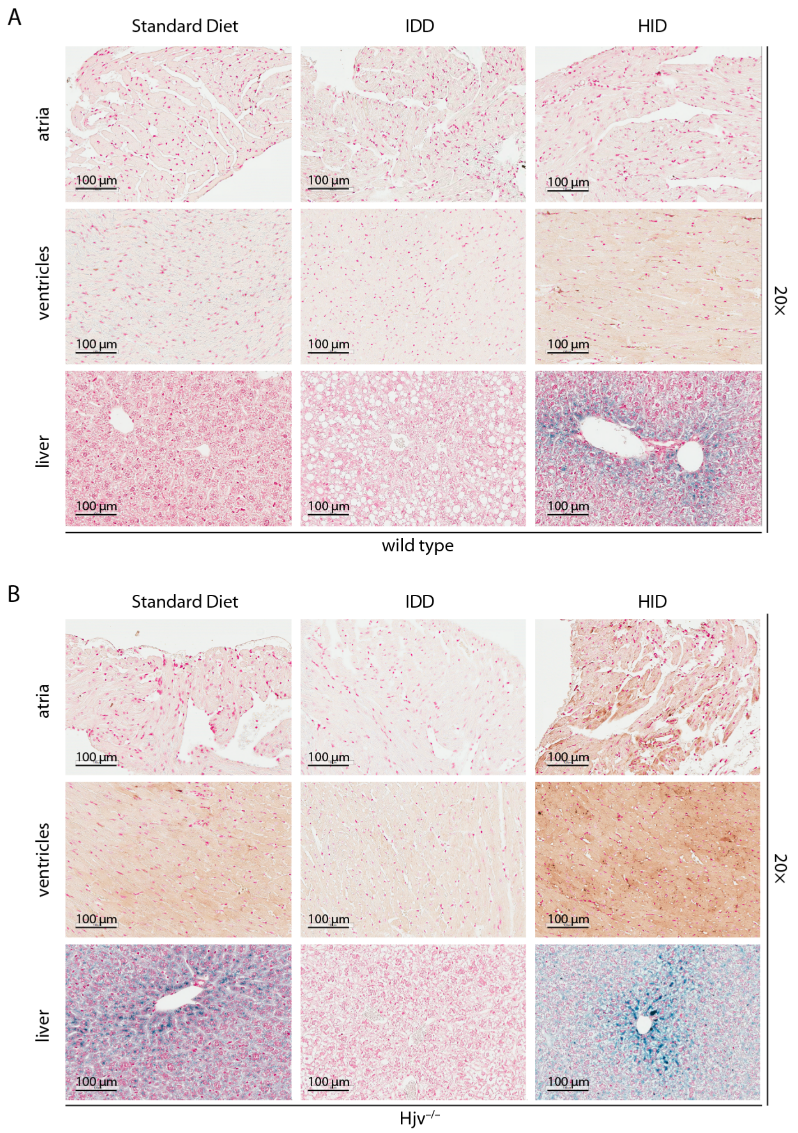

Figure S1: Perls staining of liver sections, and diaminobenzidine-enhanced Perls staining of heart atria and ventricles sections from wild type (A) and Hjv$^{-/-}$ (B) mice following dietary iron manipulations, as described in Fig. 4; magnification: 20×.

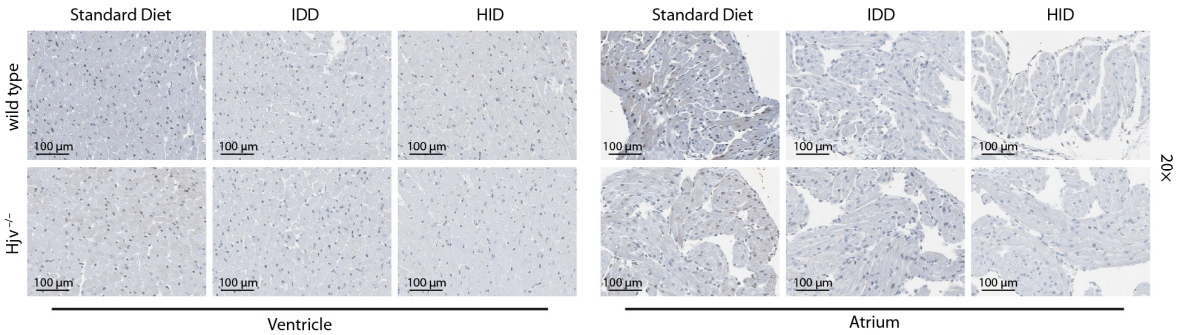

Figure S2: Immunohistochemical staining with a hepcidin antibody. Sections from heart ventricles and atria from mice subjected to dietary iron manipulations similar to those described in Fig. 4 were used for immunohistochemical detection of hepcidin; magnification: 20×.

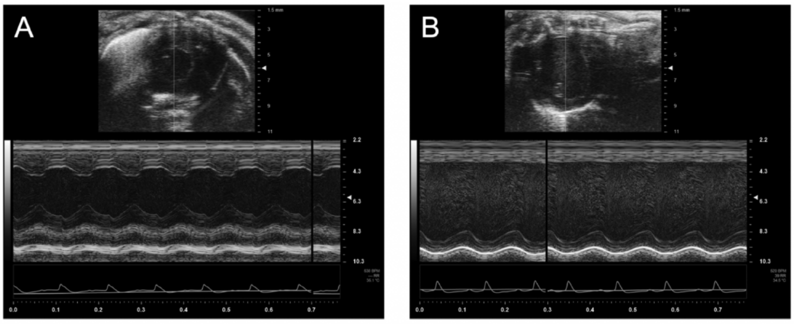

Figure S3: Representative echocardiography in a wild type mouse before (A), and 10 days after myocardial infarction surgery (B). The image shows a 2-D short-axis view of the mid left ventricle (LV) at the level of the papillary muscles.
