# Supplementary material for: Cardiac Hamp mRNA Is Predominantly Expressed in the Right Atrium and Does Not Respond to Iron
Source: Int J Mol Sci. 2023 Mar 8;24(6):5163. doi: 10.3390/ijms24065163 (PMC10049151; doi:10.3390/ijms24065163)
Supplement: Supplementary file 1 [file ijms-24-05163-s001.zip › ijms-2208971-Table S1.pdf]

Table S1. List of primers used for qPCR.

Gene	GenBank accession	Forward primer sequence	Reverse primer sequence
Hamp	NM_032541.1	*AAGCAGGGCAGACATTGCGAT*	*CAGGATGTGGCTCTAGGCTATGT*
Rpl19	NM_009078.2	*AGGCATATGGGCATAGGGAAGAG*	*TTGACCTTCAGGTACAGGCTGTG*
Acta1	NM_001272041.1	*CGTGAAGCCTCACTTCCTACC*	*CGGCTTGTTCACTTGCAGGA*
Nr2f1	NM_010151.4	*TGGCCTTCATGGACCACATC*	*TGCTCGATGACAGAGGAGGA*
Hjv	NM_027126.4	*ATCCCCATGTGCGCAGTTT*	*GCTGGTGGCCTGGACAAA*
Smad7	NM_001042660.1	*CGGACAGCTCAATTCGGAC*	*GGTAACTGCTGCGGTTGTAA*
Slc40α1(+IRE)	NM_016917.2	*GGCATAAGGCTGTTGTGCTT*	*TCATGACACCAGGCGTTCTC*
Il6	NM_031168.2	*AGCCAGAGTCCTTCAGAGAGA*	*GGTCTTGGTCCTTAGCCACTC*
HAMP	NM_021175	*ATGGCACTGAGCTCCCAGAT*	*ACTTTGATCGATGACAGCAG*
RPS18	NM_022551	*TGTGGTGTTGAGGAAAGCAG*	*AAGTGACGCAGCCCTCTATG*
SMAD7	NM_005904.4	*ACTTCTTCATGGTGTGCGGA*	*AATTGAGCTGTCCGAGGCAA*
